# Extracellular membrane vesicles—previously unrecognized components of *Staphylococcus aureus* biofilms

**DOI:** 10.1128/jb.00210-26

**Published:** 2026-08-07

**Authors:** Jinger Lei, Misaki Foster, Emery Ng, Erin S. Gloag, Xiaogang Wang

**Affiliations:** 1 Department of Biomedical Sciences and Pathobiology, Virginia-Maryland College of Veterinary Medicine, Virginia Polytechnic Institute and State University 229659, Blacksburg, Virginia, USA; 2 Center for Emerging, Zoonotic, and Arthropod-borne Pathogens, Virginia Polytechnic Institute and State University 1757, Blacksburg, Virginia, USA; 3 Fralin Life Sciences Institute, Virginia Polytechnic Institute and State University, Blacksburg, Virginia, USA; University of Illinois Chicago, Chicago, Illinois, USA

**Keywords:** *Staphylococcus aureus*, membrane vesicle, biofilms, extracellular matrix, extracellular DNA

## Abstract

*Staphylococcus aureus* is a leading cause of biofilm-associated infections, in which communities of bacterial cells are encased in an extracellular matrix composed of polysaccharides, proteins, and extracellular DNA (eDNA) that protect bacteria from host immune defense and antibiotics. Despite their importance, the mechanisms by which matrix components are released from bacterial cells and incorporated into the biofilm matrix remain poorly understood. Using a drip-flow biofilm system, we showed that membrane vesicles (MVs) were associated with the biofilm matrix formed by *S. aureus* clinical isolate MN8. Proteomic analysis of biofilm matrix proteins and purified MVs showed that biofilm-derived MVs carried cytoplasmic, membrane, and extracellular proteins that closely resembled the protein composition of the biofilm matrix, but differed significantly from MVs produced by planktonic cultures. Biofilm-derived MVs carried significantly higher levels of DNA than MVs from planktonic cultures, and MV-associated DNA was sensitive to DNase treatment, suggesting that eDNA is primarily associated with the MV surface. Although strain MN8 is known to form polysaccharide-dependent biofilms, exogenously added DNase or proteinase K significantly impaired biofilm formation and integrity. Importantly, supplementation with biofilm-derived MVs, but not MVs from planktonic cultures, significantly restored biofilm formation in enzyme-treated static cultures. Together, these findings provide evidence that *S. aureus* MVs are generated within biofilms, and that these MVs serve as an important resource of matrix components and contribute to biofilm formation.

**IMPORTANCE:**

Extracellular membrane vesicles (MVs) are important mediators of intercellular communication and have been implicated in the bacterial physiology and pathogenesis. MVs in fungi and gram-negative bacteria mediate key biofilm processes, such as formation and structural maintenance. However, MV production and function in biofilm formation in gram-positive bacteria have remained largely unexplored. Here, we report for the first time the purification and characterization of MVs derived from *Staphylococcus aureus* biofilms. Our studies demonstrate that *S. aureus* MVs are important components of the biofilm matrix that contribute to biofilm formation by serving as carriers of key matrix components. This work advances our limited understanding of MVs in gram-positive bacteria and reveals a previously unrecognized mechanism contributing to *S. aureus* biofilm formation.

## INTRODUCTION

*Staphylococcus aureus* is a leading cause of biofilm-associated infections (1), including those related to medical implants and osteomyelitis, both of which present significant clinical challenges (2, 3). Biofilms are structured community of microbial cells encased within a self-produced extracellular matrix, which plays a key role in chronic infections by protecting bacteria from both the immune system and antibiotics (4). The biofilm extracellular matrix (ECM) is comprised of proteins, polysaccharides, and extracellular DNA (eDNA). Although the production of poly-N-acetyl-β- (1–6)-glucosamine (PNAG), also known as polysaccharide intercellular adhesion, is required for biofilm formation in some *S. aureus* strains, particularly methicillin-sensitive *S. aureus*, most methicillin-resistant *S. aureus* (MRSA) isolates rely primarily on proteins rather than polysaccharides for biofilm formation (5–8). Recent proteomic analyses of *S. aureus* biofilm ECMs have revealed that both protein- and polysaccharide-dependent biofilms share a core exoproteome enriched in cytoplasmic proteins (9–12), which contribute to biofilm formation by moonlighting as ECM components. These proteins, together with extracellular DNA (eDNA), help maintain biofilm integrity through a complex network of electrostatic interactions (13, 14). More recently, this model has been extended to include lipoproteins that function as anchor points between eDNA and the bacterial cell surface (15). Despite significant progress in characterizing ECM components, the mechanisms governing the release and incorporation of matrix components, and the forms in which they are present within the biofilm matrix remain poorly understood.

Extracellular membrane vesicles (MVs) are nano-sized, spherical particles that are produced by eukaryotes, archaea, and bacteria (16). MVs have been recognized as important cargo carriers in *S. aureus* planktonic cultures (17–25), encapsulating cytoplasmic, membrane, and extracellular proteins, as well as glycopolymers and nucleic acids (17, 21–23, 26–28). MVs generated from *S. aureus* planktonic cultures induce cytotoxicity in various cell types (23, 29–31), elicit pro-inflammatory responses (21, 25, 31–34), and promote bacterial survival in human and animal blood (34). Moreover, *S. aureus* MVs have been detected during *in vivo* acute infection (35), shedding light on their physiological relevance in disease settings. Whereas these findings suggest a role of MVs in *S. aureus* virulence, whether MVs are produced in staphylococcal biofilms and contribute to biofilm biology remain largely unexplored.

Biofilms represent a sessile, matrix-embedded lifestyle underlying many chronic and device-related infections, where bacteria persist despite activated immune defense and antibiotic treatments. These distinct conditions may profoundly influence MV production, composition, and function. MVs produced within *Candida albicans* biofilms carry specific cargo proteins that contribute to the development of biofilms and their drug resistance (36–39). In addition, MVs from *C. albicans* biofilms cultivated for different time points exhibit temporal differences in inducing host cell responses (40). In gram-negative bacteria, outer membrane vesicles (OMVs) have been characterized in biofilms produced by multiple bacterial species, such as *Pseudomonas aeruginosa* (41)*, Vibrio cholerae* (42)*, and Helicobacter pylori* (43)*,* and these OMVs play a variety of roles in biofilm development (44). For example, OMV production is induced by quinolone signal in *P. aeruginosa* biofilms, and these OMVs promote biofilm dispersal through encapsulation of enzymes capable of degrading matrix components (45). Conversely, OMVs generated within *V. cholerae* biofilms contribute to biofilm matrix assembly by carrying key matrix proteins and polysaccharide (42).

Although considerable progress has been made in understanding the role of MVs in biofilm formation across diverse microbes, their production, composition, and function in gram-positive bacteria remain poorly characterized. *Bacillus subtilis* was shown to produce MVs in *in vitro* biofilms, but the cargo of these MVs and their role in biofilm formation remain unexplored (46). Two studies visualized MVs in electron micrographs of static *S. aureus* biofilms grown in tissue culture plates, but MVs were neither purified nor functionally characterized (47, 48). In this study, we provide the first characterization of MV production within *S. aureus* biofilms, utilizing a well-established drip-flow biofilm system. We provide evidence that *S. aureus* MVs are important carriers of key matrix components, including eDNA and proteins, and contribute to biofilm formation. Our study provides new insights into the roles of MVs in biofilm formation in gram-positive bacteria.

## RESULTS

### Purification of MVs from *S. aureus* biofilms using a drip-flow biofilm system

**Fig 1:**
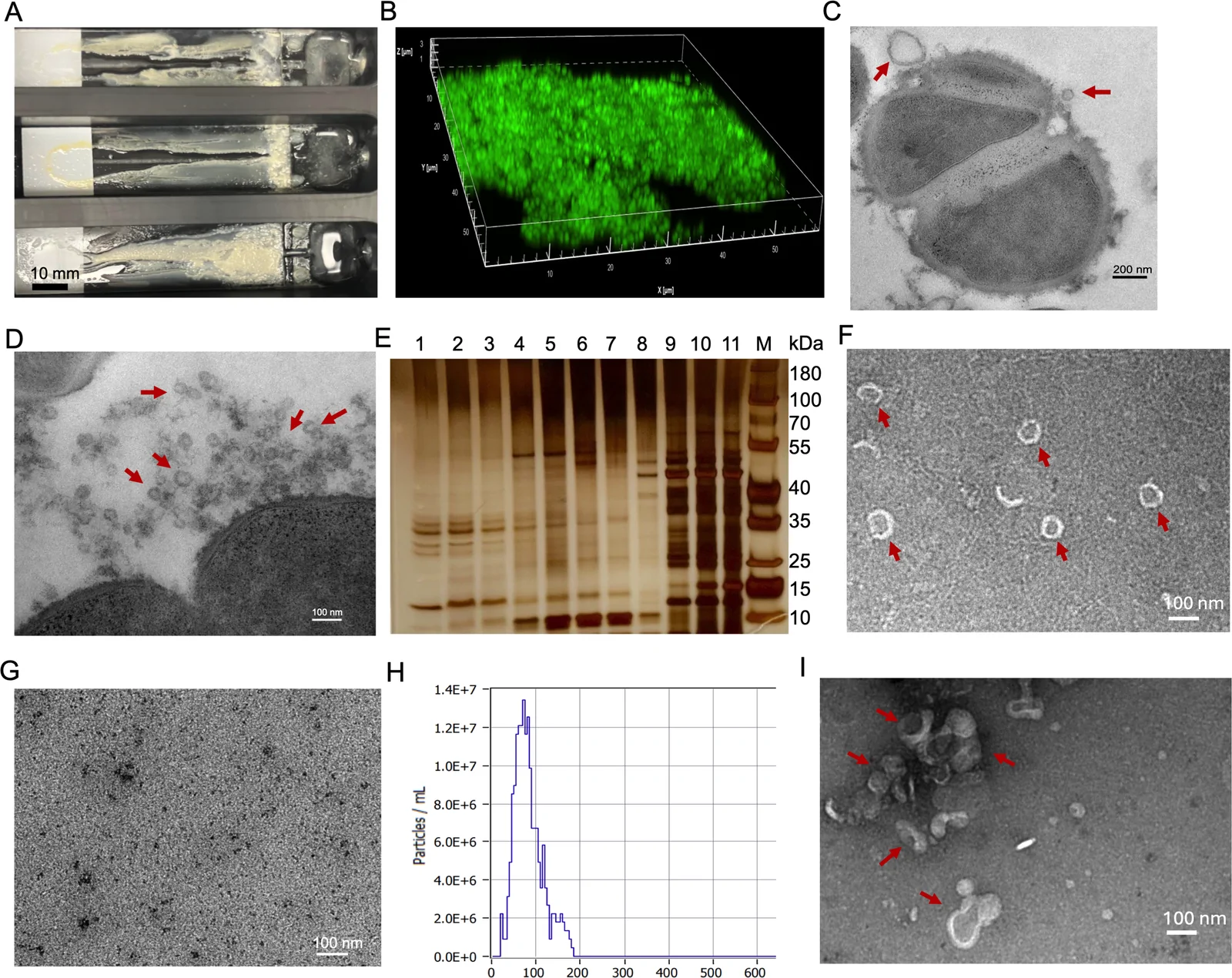
Generation of MVs in *S. aureus* MN8 drip-flow biofilms. (**A**) *S. aureus* MN8 biofilms formed on glass slides (25 mm × 75 mm) using a drip-flow bioreactor. (**B**) Representative fluorescent image of MN8 drip-flow biofilms stained with SYTO-9 DNA dye. (**C and D**) Ultrathin sections of MN8 drip-flow biofilms examined by TEM revealed MVs (indicated by red arrows) released from the cell (**C**) or present in the matrix (**D**). (**E**) Silver staining of fractions recovered from the density gradient ultracentrifugation. (**F–G**) TEM showed purified MVs (indicated by red arrows) in pooled fractions 1 to 7 (**F**), but not in fractions 8–11 (**G**). (**H**) The size distribution of purified MVs was measured by a nanoparticle tracking analyzer. (**I**) MVs (indicated by red arrows) purified from effluent medium of drip-flow biofilms were imaged by TEM.

We used a drip-flow biofilm system (49–52) to cultivate *S. aureus* MN8 biofilms at 37°C. This system allows biofilm growth under low shear at the air-liquid interface, promoting high biomass. *S. aureus* MN8 biofilms formed on glass slides by 48 h (Fig. 1A), and biofilms stained with the DNA dye SYTO-9 were imaged with confocal laser scanning microscopy (Fig. 1B). To assess the presence of *S. aureus* MVs in biofilms, biofilms were harvested, and ultrathin sections of biofilms were examined by transmission electron microscopy (TEM). We observed MV release from the *S. aureus* cell surface (Fig. 1C), and massive numbers of vesicles were present in the biofilm matrix (Fig. 1D).

Crude MVs from the filter-sterilized supernatants of biofilm homogenates were pelleted by ultracentrifugation and subjected to OptiPrep density gradient ultracentrifugation to remove membrane fragments and protein aggregates. Consecutive OptiPrep fractions (20 µL) were subjected to SDS-PAGE, and the proteins were silver stained. OptiPrep fractions with similar protein profiles (fractions 1–7 and 8–11; Fig. 1E) were pooled and diafiltrated in PBS. TEM imaging confirmed the presence of MVs in pooled fractions 1–7 (Fig. 1F), but not in fractions 8–11 (Fig. 1G). Nanoparticle Tracking Analysis (NTA) revealed a recovery yield of approximately nine particles per CFU from the biofilm matrix, and purified MVs showed an average size of 80 nm (Fig. 1H).

To determine whether MVs were also shed from the biofilm matrix during biofilm growth, the effluent medium from drip-flow biofilms was collected after 24 h and filter-sterilized. MVs pelleted by ultracentrifugation were further purified by OptiPrep density gradient ultracentrifugation. The recovery of MVs from effluent medium was confirmed by TEM imaging (Fig. 1I), indicating that MVs were also shed into the medium from *S. aureus* biofilms.

### Proteomic analysis of *S. aureus* MN8 biofilm matrix and biofilm-derived MVs

**Fig 2:**
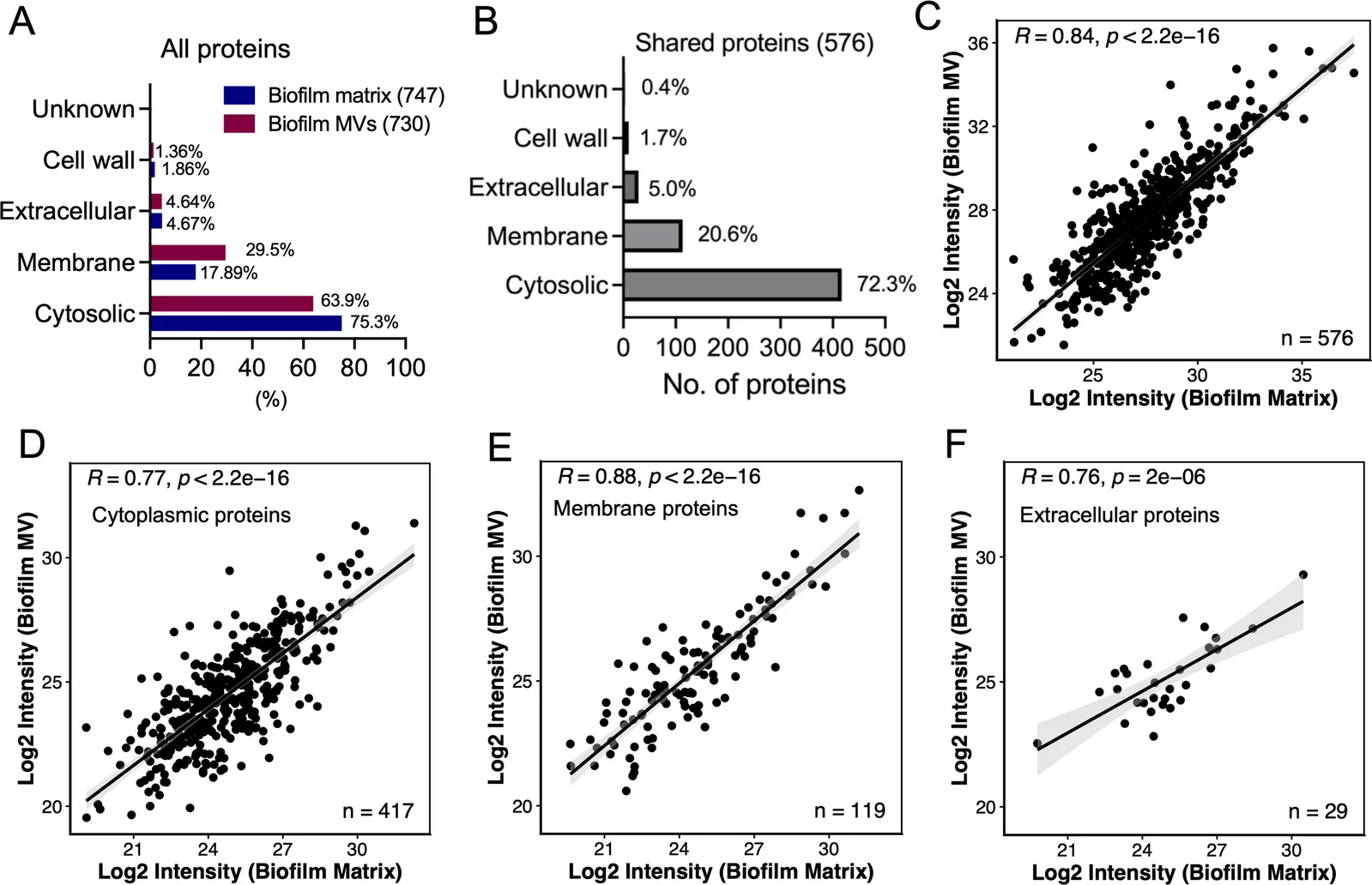
Proteomic analysis of MVs purified from *S. aureus* MN8 drip-flow biofilms and the biofilm matrix. (**A**) Subcellular localization of total proteins identified from purified MVs and the biofilm matrix. (**B**) Subcellular localization of proteins shared by purified MVs and the biofilm matrix. (**C–F**) Correlation of protein abundance between MVs and the biofilm matrix for proteins shared between two groups. Plots show correlations for (**C**) all shared proteins, (**D**) cytoplasmic proteins, (**E**) cell membrane proteins, and (**F**) extracellular proteins. Distributions are shown with linear regression lines.

We analyzed the protein contents of MN8 biofilm matrices, as well as MVs purified from MN8 drip-flow biofilms, and MVs from planktonic cultures, using liquid chromatography-tandem mass spectrometry (LC-MS/MS). The *S. aureus* MN8 chromosome is 2,882,664 bp in size and comprises 2,833 predicted protein-coding sequences. This includes 1,360 cytoplasmic proteins, 715 cytoplasmic membrane proteins, 40 cell wall-associated proteins, 98 extracellular proteins, and 619 proteins with unknown subcellular localization. We identified 747 proteins in the biofilm matrix and 730 proteins in MVs purified from biofilms (Table S1). Subcellular localization predictions indicated that most proteins in both the biofilm matrix and the biofilm-derived MVs were classified as cytoplasmic, followed by membrane-associated, extracellular, and cell wall-associated proteins (Fig. 2A). Of the 56 predicted lipoproteins in the MN8 genome, 31 were detected in the biofilm matrix and 35 were in biofilm-derived MVs (Table S2). Five hundred seventy-six proteins were shared between the biofilm matrix and biofilm-derived MVs (Table S3). Among the shared proteins, 417 proteins (72.3%; Fig. 2B) were cytosolic, representing 74.4% and 89.3% of cytosolic proteins identified in the biofilm matrix and biofilm-derived MVs, respectively. 20.6% of shared proteins were membrane-associated (Fig. 2B), representing 83.6% of membrane proteins identified in the biofilm matrix. Twenty-nine of 576 (5.0%) shared proteins were extracellular proteins (Fig. 2B), representing 83% and 85% of extracellular proteins present in the biofilm matrix and biofilm-derived MVs, respectively. Many of these extracellular proteins are virulence factors, such as leukocidins LukAB and HlgBC, β-type phenol-soluble modulin (PSMβ1), chemotaxis inhibitory protein CHIPS, and proteases staphopain A (ScpA) and B (SspB).

Due to the high similarity between the protein contents of biofilm-derived MVs and the biofilm matrix, we hypothesized that the relative abundance of proteins in the biofilm matrix dictates their incorporation into biofilm-derived MVs. To test this hypothesis, we performed a correlation analysis of 576 shared proteins between the two groups, comparing the relative abundance of individual proteins in the biofilm matrix with their abundance in biofilm-derived MVs. Similar correlation coefficients were observed when all MV-associated proteins were analyzed together, as well as when cytoplasmic, membrane, and extracellular proteins were analyzed separately (Fig. 2C through F).

### Comparison of MV proteomes from biofilm and planktonic cultures

**Fig 3:**
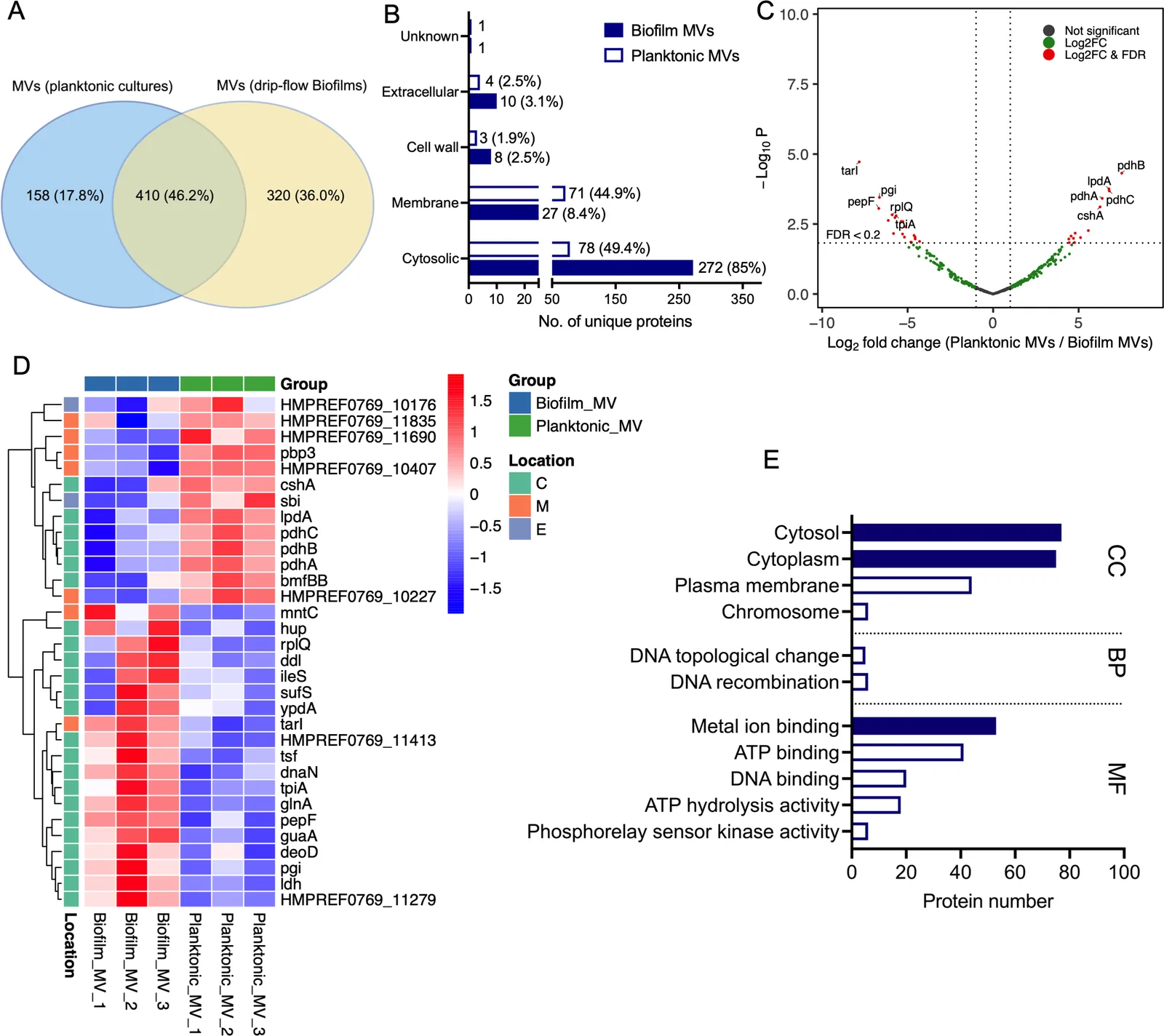
Comparison of the proteome of MVs derived from *S. aureus* MN8 biofilms and planktonic cultures. (**A**) The Venn diagram illustrates the shared and unique proteins between MVs isolated from drip-flow biofilms and planktonic cultures. Numbers within the diagram sections indicate the count and percentage of unique and shared proteins between the two groups. (**B**) Subcellular localization of unique proteins present in biofilm-derived MVs or MVs from planktonic cultures. (**C**) Volcano plot shows the differential abundance of 410 shared proteins in MVs produced from biofilms and planktonic cultures. Each dot represents a protein, with the x-axis showing the log2 fold change (Log2FC) in protein intensities between biofilm-derived MVs and MVs from planktonic cultures. The y-axis shows the −log10 *P* value of the change in intensity. Proteins with significant differential abundance at FDR < 0.2 (adjusted *P* value) are highlighted in red color. Ten of the proteins showing the greatest differential enrichment between biofilm-derived MVs and MVs from planktonic cultures are labeled with their gene names. (**D**) Heatmap view of 32 proteins with differential abundance (FDR < 0.2) among the shared proteins between the two groups. Each column indicates a biological replicate, and each row represents a protein. Predicted subcellular localizations are indicated in the legend. Proteins within each localization group were hierarchically clustered. (**E**) Gene ontology over-representation analysis of GO terms enriched among differentially abundant and unique proteins in biofilm-derived MVs (filled blue bars) and MVs from planktonic cultures (empty bars). The y-axis shows GO terms categorized into cellular component (CC), biological process (BP), and molecular function (MF), while the x-axis indicates the number of proteins annotated to each enriched GO term. Only GO terms with BH adjusted *P* < 0.05 are shown.

Biofilms and planktonic cultures represent two distinct bacterial growth modes. We analyzed the protein cargo of MVs purified from MN8 planktonic cultures and compared it with that of biofilm-derived MVs. In total, 568 proteins were identified in MVs purified from MN8 planktonic cultures (Table S1), compared with the 730 proteins identified in biofilm-derived MVs. Among the planktonic MV proteins, 48.1% were cytoplasmic, 45.3% were membrane-associated, 4.91% were extracellular, and 0.87% were cell wall-associated. Four hundred ten proteins were shared by MVs from both culture conditions, but 320 and 158 unique proteins were identified exclusively in biofilm- and planktonic-derived MVs, respectively (Fig. 3A). Among the 320 unique proteins identified in biofilm-derived MVs, 272 were cytosolic proteins; the remaining proteins were membrane-associated (27), extracellular (10), and cell wall-associated proteins (8) (Fig. 3B). In contrast, 78 and 71 unique proteins present in MVs derived from planktonic cultures were cytoplasmic and membrane-associated proteins, respectively (Fig. 3B). Notably, several proteins involved in the survival of *S. aureus* under stress conditions, including catalase, alkyl hydroperoxide reductase C, acetolactate synthase, thioredoxin, thioredoxin reductase, and thiol peroxidase, were only identified in biofilm-derived MVs.

To further compare the shared proteins between the two groups, we performed a volcano plot analysis using a fold change threshold of 2.0 (Fig. 3C). The analysis revealed 32 differentially abundant proteins showing significant differences (False discovery rate [FDR] < 0.2) between the biofilm-derived MVs and MVs from planktonic cultures. Among these, 19 proteins were more abundant in biofilm-derived MVs, while 13 had higher levels in the MVs from planktonic cultures (Fig. 3D). Gene ontology (GO) over-representation analysis of differentially abundant and unique proteins revealed that cytoplasmic proteins and proteins involved in metal ion binding were enriched in biofilm-derived MVs (Fig. 3E). In contrast, membrane-associated proteins were enriched in MVs from planktonic cultures. Additionally, molecular function and biological process analyses indicated that proteins involved in DNA binding, ATP binding, and DNA recombination were enriched in MVs from planktonic cultures (Fig. 3E). Together, these analyses demonstrate that *S. aureus* MN8 biofilms produce MVs with a protein cargo that is compositionally distinct from those generated under planktonic conditions.

### Analysis of extracellular DNA associated with biofilm-derived MVs

**Fig 4:**
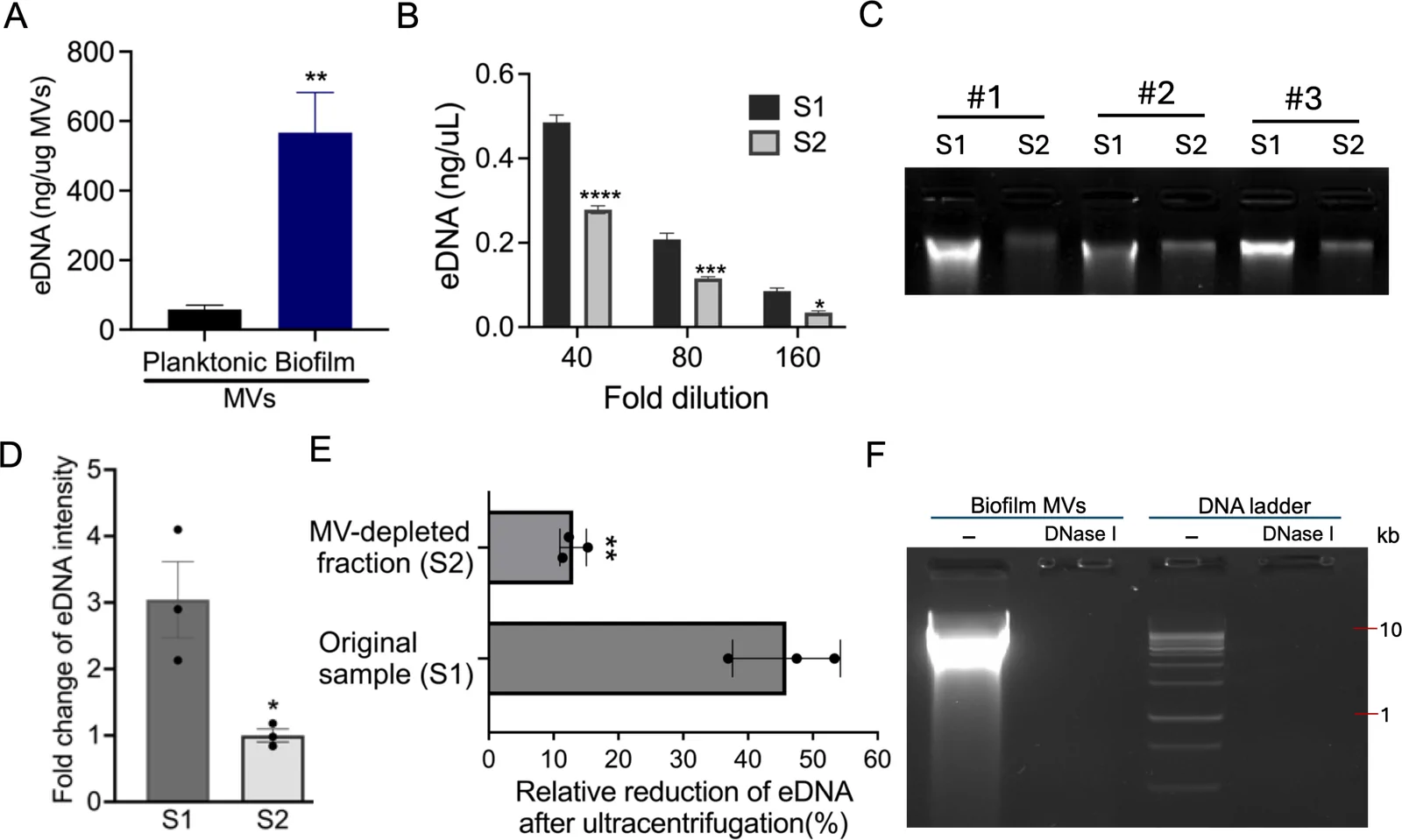
Association of eDNA with MVs derived from *S. aureus* MN8 biofilms. (**A**) Quantification of eDNA associated with MN8 MVs purified from drip-flow biofilms or planktonic cultures. (**B**) Quantification of eDNA present in serially diluted biofilm homogenates before (S1) and after (S2) MV depletion, measured using an eDNA quantification kit with a DNA standard curve. (**C**)Before (S1) and after (S2) MV depletion, 30 μL aliquots of filter-sterilized biofilm homogenates were loaded onto a DNA agarose gel and stained with SYBR Safe DNA dye. Samples from three independent experiments were analyzed. (**D**) Quantification of DNA signal intensities for S1 and S2 from triplicate samples on a DNA agarose gel. (**E**) Relative reduction of eDNA following ultracentrifugation of the original biofilm samples (S1) and the MV-depleted fraction (S2), respectively. (**F**) Biofilm-derived MVs (bMVs) and 1 kb DNA ladder were treated for 1 h with or without 100 μg/mL DNase I, followed by agarose gel electrophoresis and staining with SYBR Safe DNA dye. Data are presented as mean ± standard error of the mean and analyzed using the Student’s *t-*test. *P < 0.05, **P < 0.01, ***P < 0.001, ****P < 0.0001.

When we compared eDNA associated with biofilm-derived MVs with that associated with MVs from planktonic cultures, the former carried significantly higher levels of eDNA than MVs from planktonic cultures (Fig. 4A).

We next measured eDNA present in MN8 biofilm homogenates before (S1) and after depletion of MVs (S2) by ultracentrifugation using a highly sensitive dsDNA quantification kit (13). Depletion of MVs significantly reduced the eDNA content of biofilm homogenates (Fig. 4B). In parallel, equal volumes of S1 and S2 fractions were subjected to agarose gel electrophoresis, and DNA was stained with SYBR Safe DNA dye. Again, S2 fractions (MV-depleted) showed markedly lower DNA levels than S1 samples (Fig. 4C and D). To assess whether free eDNA was pelleted during ultracentrifugation, MV-depleted supernatants (S2) were subjected to a second round of ultracentrifugation under the same conditions used for MV isolation. Ultracentrifugation of MV-depleted samples resulted in only a ~12% reduction in eDNA relative to its level in the original biofilm samples, compared with the approximately 50% reduction observed after the initial ultracentrifugation of original samples (S1) (Fig. 4E). These results suggest that most of the eDNA removed during the initial ultracentrifugation was associated with the MVs, while the majority of free eDNA remaining in the supernatant was not pelleted under the same conditions.

We further treated biofilm-derived MVs for 1 h with 100 µg/mL DNase I before analysis by agarose gel electrophoresis. Both MV-associated eDNA and the DNA ladder control were degraded by DNase I (Fig. 4F). Collectively, these data indicate that a substantial proportion of eDNA in the *S. aureus* biofilm matrix is associated with MVs, and MV-associated eDNA is sensitive to nuclease treatment.

### Detection of glycopolymers in MVs produced from *S. aureus* biofilms

**Fig 5:**
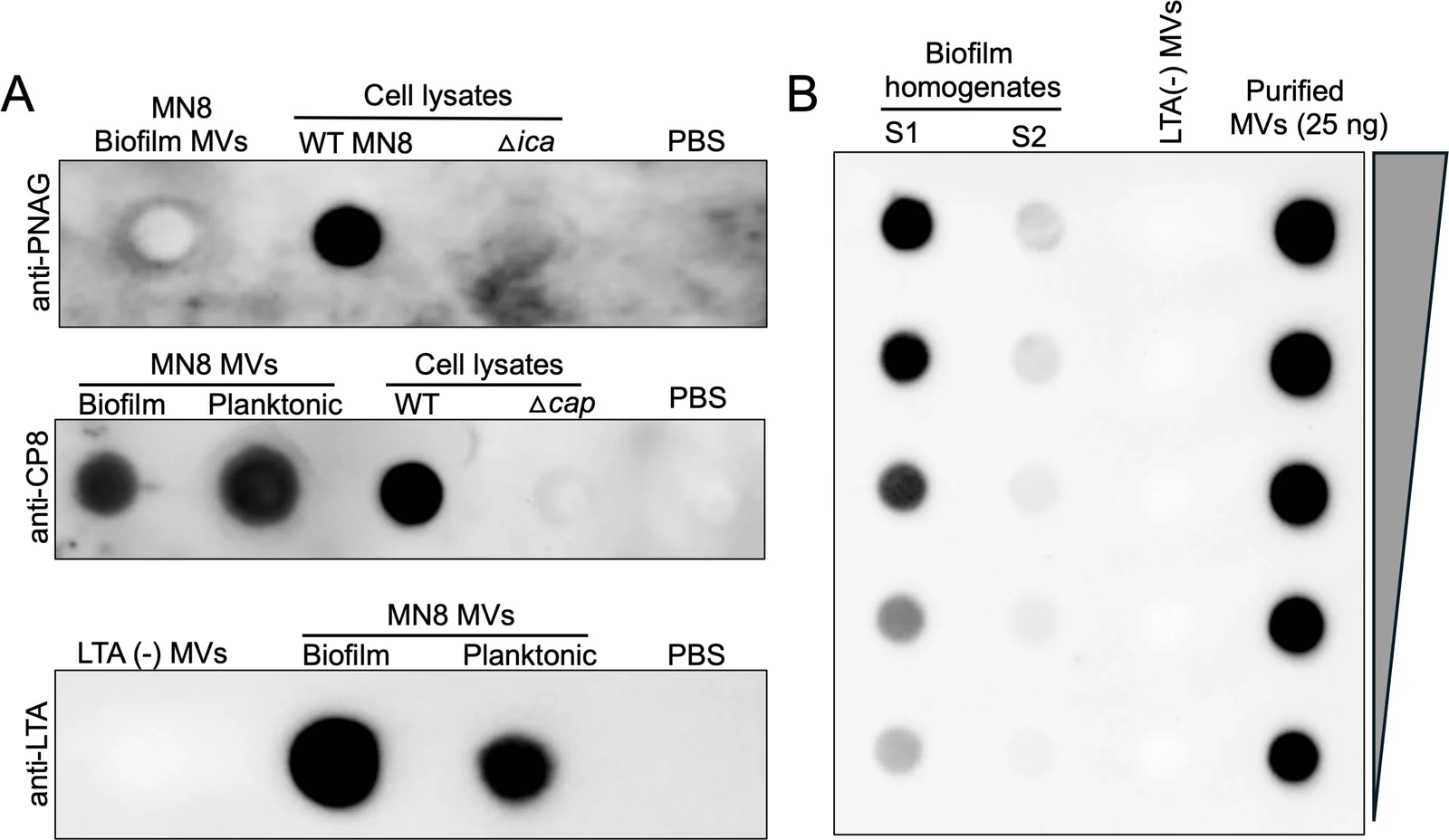
Detection of glycopolymers associated with *S. aureus* MN8 MVs. (**A**) The presence of PNAG, CP8, or LTA in MVs derived from biofilms or planktonic cultures was determined by dot blots using specific antibodies. Where indicated, cell lysates from WT MN8 or relevant mutants deficient in PNAG or CP8 production were used as positive or negative controls. For LTA, MVs produced from an LTA-deficient *S. aureus* strain served as a control. PBS was included as a loading control. (**B**) A total of 100 μL of two-fold serial dilutions of filter-sterilized biofilm homogenates or MVs (25 ng) purified from biofilms were applied to nitrocellulose membranes, and LTA was detected using anti-LTA antibodies. Serial dilutions of LTA-negative (–) MVs (2.5 µg) were used as a negative control. All experiments were repeated at least twice with similar results.

Because PNAG is a major component of *S. aureus* MN8 biofilms, we examined the association of PNAG with MVs by immunodot blot using human antibodies against PNAG (53). PNAG was detected in WT MN8 cell lysates, but not in either biofilm-derived MVs or MN8Δ*ica* cell lysates (Fig. 5A), which served as negative controls. *S. aureus* MN8 produces type eight capsular polysaccharide (CP8). We found that CP8 was associated with MVs derived from both biofilm and planktonic cultures (Fig. 5A). As controls, CP8 was present in WT MN8 cell lysates but absent in the capsule mutant (Δ*capHIJK*) lysates (Fig. 5A).

Lipoteichoic acid (LTA) is a membrane-anchored glycopolymer that is highly abundant in MVs produced from planktonic cultures (17). LTA was detected in as little as 25 ng of MVs purified from either biofilms or planktonic cultures (Fig. 5A). In contrast, no signal was observed with up to 2.5 μg of MVs produced from an LTA-deficient *S. aureus* strain (54), confirming the specificity of the assay. To determine whether LTA in the biofilm matrix was MV-associated, we depleted MVs from biofilm homogenates by ultracentrifugation. Dot blot analysis of 2-fold diluted samples showed the presence of LTA in original biofilm homogenates and purified MVs, but not in MV-depleted homogenates (Fig. 5B). Together, these results indicate that LTA molecules present in MN8 biofilms are predominantly associated with MVs.

### Functional role of *S. aureus* MVs in biofilm formation

**Fig 6:**
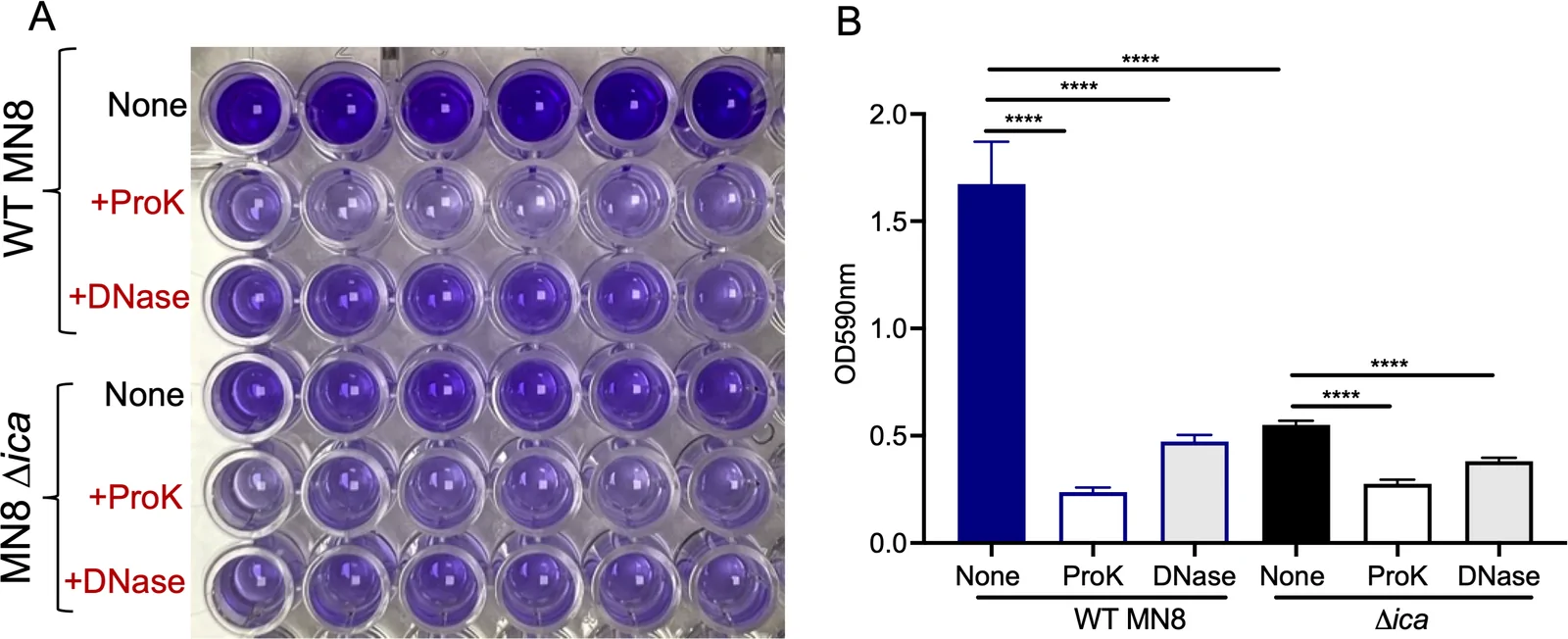
Proteins and eDNA are required for *S. aureus* MN8 biofilm formation. (**A**) *S. aureus* MN8 and MN8Δ*ica* biofilms were grown for 24 h in TSB supplemented with 0.2% glucose and 1% NaCl, followed by incubation for an additional 24 h in fresh medium with or without DNase I (100 µg/mL) or proteinase K (20 µg/mL). Biofilms were washed three times with PBS before staining with 0.2% crystal violet solution. (**B**) Biofilm biomass was quantified by measuring the absorbance of the stained biofilms at 590 nm. Data are presented as mean ± SEM, and statistical analyses were performed using one-way ANOVA with Dunnett’s multiple comparison test. ****P  <  0.0001.

Recent studies have suggested that both eDNA and proteins remain important for biofilm formation in PNAG-positive *S. aureus* strains (10, 14, 15, 55). Strain MN8 is well known for its PNAG-dependent biofilms, and eDNA is released during biofilm formation (55). To evaluate the contribution of these components to biofilm formation, we cultivated WT MN8 and its PNAG-negative strain ∆*ica* for 24 h in tissue culture plates containing tryptic soy broth (TSB) supplemented with 0.2% glucose and 1% NaCl. The resulting biofilms were then incubated for an additional 24 h in fresh medium containing either 20 µg/mL proteinase K or 100 µg/mL DNase I. Biofilm formation was quantified using crystal violet staining. As expected, the MN8∆*ica* mutant formed substantially less biofilm than the WT strain, confirming the role of PNAG in MN8 biofilm formation. Of note, both DNase I and proteinase K treatments resulted in significant reductions in biofilm biomass in both WT MN8 and the ∆*ica* mutant (Fig. 6A and B). Overall, these results demonstrate that PNAG, eDNA, and proteins are all key components of MN8 biofilms and contribute to their structural integrity.

**Fig 7:**
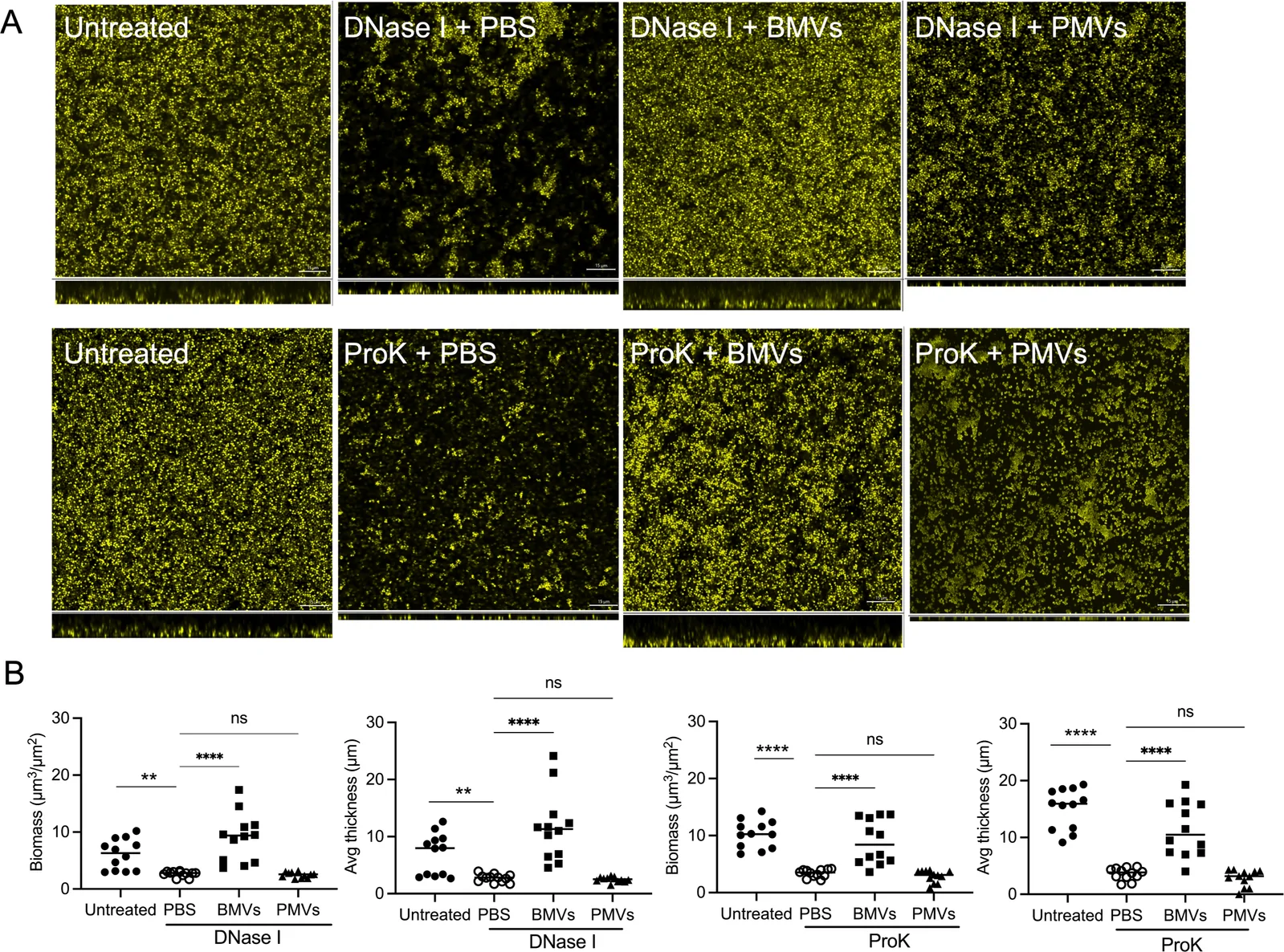
Biofilm-derived MVs promote biofilm formation. (**A and B**), 10 μg/mL of MVs purified from drip-flow biofilms (BMVs) or planktonic cultures (PMVs) were added to 24-h MN8 biofilms that were pretreated for 6 h with proteinase K (20 μg/mL) or DNase I (100 μg/mL), followed by another 24 h incubation. Biofilms of each treatment were imaged by confocal microscopy (**A**), and biofilm thickness and biomass were quantified from 12 images obtained from three biological replicates performed on different days using three independent batches of purified MVs (**B**). Data are presented as mean ± SEM, and statistical analyses were performed using one-way ANOVA with Tukey’s multiple comparisons test. *P  <  0.05, **P  <  0.01, ***P  <  0.001, ****P  <  0.0001.

Because MVs carry a substantial amount of eDNA and proteins present in MN8 biofilms, we evaluated whether MVs contribute to biofilm formation. 10 µg/mL of MVs purified from biofilms or planktonic cultures were added to 24-h MN8 static biofilms that had been pretreated with DNase I or proteinase K, followed by an additional 24 h incubation. Biofilm formation was then assessed by confocal microscopy. Addition of biofilm-derived MVs, but not MVs from planktonic cultures or PBS, significantly restored biofilm formation in static cultures pretreated with DNase I or proteinase K, as evidenced by increased biofilm biomass and biofilm thickness (Fig. 7A and B). To confirm whether the MV-associated eDNA and proteins are responsible for these effects, biofilm-derived MVs were pretreated with either DNase I or proteinase K for 1 h before being added to static cultures pretreated with the corresponding enzyme. As expected, only untreated MVs restored biofilm biomass and thickness, whereas DNase I- or proteinase K-treated MVs failed to restore biofilm formation (Fig. S1A and B). Together, these results suggest that eDNA and proteins associated with biofilm-derived MVs serve as important structural components of *S. aureus* biofilms and contribute to biofilm formation.

*S. aureus* phenol-soluble modulins (PSMs) are positively regulated by the Agr system and contribute to biofilm dispersal once their protein levels reach a critical threshold (56). Although PSM-mediated dispersal is thought to involve disruption of non-covalent interactions within the biofilm matrix (56), the molecular details remain unclear. Because MVs are enriched in matrix proteins and eDNA and likely play a critical role in matrix formation, it is plausible that PSM-mediated biofilm dispersal occurs, at least in part, through disruption of biofilm-associated MVs. To test this hypothesis, we compared MV production between WT MW2 and its Δ*psmα*/*β*/*hld* mutant in drip-flow biofilms. As expected, the Δ*psmα*/*β*/*hld* mutant formed substantially thicker biofilms than the WT MW2 after 48 h of growth in the drip-flow system (Fig. S2A). MVs isolated from both WT and mutant biofilms were quantified by NTA and normalized to bacterial CFUs recovered from each biofilm. Strikingly, more than 5-fold higher MV levels were recovered from Δ*psmα/β/hld* biofilms (Fig. S2B).

## DISCUSSION

Recent studies have shown that MVs produced by both gram-negative bacteria and fungi play important roles in biofilm development, including matrix assembly, biofilm maturation, and modulation of host responses (36, 38–42, 45). In contrast, the generation and function of MVs within biofilms formed by gram-positive bacteria, such as *S. aureus,* remain largely unexplored. We provide the first characterization of MVs derived from *S. aureus* biofilms. Our results indicate that *S. aureus* MVs produced during biofilm formation are not merely trapped in biofilms but instead function as active contributors to biofilm formation. This work advances our limited understanding of the role that MVs play in biofilm formation in gram-positive bacteria.

MN8 is known to form PNAG-dependent biofilms, as disruption of the *icaADBC* operon responsible for PNAG synthesis impairs biofilm formation (57). We showed that proteinase K and DNase I treatments also significantly impaired MN8 biofilm formation, indicating that both proteins and eDNA are also important structural components of MN8 biofilms. Recent reports indicate that both eDNA and proteins are present within PNAG-dependent biofilms, and cytoplasmic proteins have been implicated in moonlighting functions within the biofilm matrix by interacting with negatively charged eDNA to contribute to biofilm integrity (10, 14, 55, 58, 59). Our proteomic analysis revealed 562 cytoplasmic proteins in the MN8 biofilm matrix. Many previously identified moonlighting proteins (10) were detected, such as glyceraldehyde-3-phosphate dehydrogenase, enolase, transketolase, and lactate dehydrogenase. In addition, we identified 131 membrane-associated proteins, including 31 lipoproteins, such as MntC, FhuD2, PrsA, and SaeP. Although the contribution of lipoproteins to *S. aureus* biofilms remains ill-defined, lipoproteins mediate TLR2-mediated immune activation, and biofilm infections trigger host inflammatory responses (60–63). Moreover, several lipoproteins identified in this work, including PrsA and SaeP, were previously shown to bind eDNA within the *S. aureus* LAC biofilm matrix (15), suggesting a dual role in biofilm structural integrity and host immune activation. Collectively, our findings provide new evidence that incorporation of cytoplasmic proteins and lipoproteins into the extracellular matrix also occurs in PNAG-dependent biofilms, suggesting a conserved strategy used by *S. aureus* to assemble the biofilm matrix.

We previously reported that *S. aureus* MVs produced in planktonic cultures serve as a secretory system enabling bacteria to transport their protected cargo to the environment and host cells (17, 21, 23, 64). Whether these findings extend to MVs generated within biofilms is unclear, as the bacterial cells cultivated under the two conditions differ substantially in physiology, composition, and immune dynamics. In this study, we purified and characterized MVs from *S. aureus* biofilms grown in a drip-flow bioreactor. NTA analysis estimated that approximately 9 MV particles were recovered per CFU from the 48-h biofilm matrix. However, this value likely underestimates the actual number of MVs produced within the biofilm matrix due to losses during purification or because they remain within the matrix. In this study, we first isolated MVs from homogenized biofilm samples, followed by further purification using OptiPrep density gradient ultracentrifugation. However, density gradient ultracentrifugation, although capable of achieving high MV purity, has been reported to yield lower recovery than other isolation methods (65). Further studies are needed to optimize methods for purifying MVs from biofilms.

Notably, 77% of proteins present in the MN8 biofilm matrix were identified in biofilm-associated MVs, and the intensity-based correlation analysis also revealed a strong positive correlation between proteins detected in biofilm-derived MVs and the biofilm matrix. Of the 562 cytoplasmic proteins identified in the MN8 biofilm matrix, 418 were enriched in biofilm-derived MVs, accounting for 89.3% of cytoplasmic proteins identified in biofilm-derived MVs. Over 80% of membrane and extracellular proteins identified in the MN8 biofilm matrix were also present in biofilm-derived MVs, including lipoproteins and toxins, such as LukAB, HlgCB, PSMβ1, and TSST1. These results suggest that MVs contribute substantially to the protein composition of the *S. aureus* biofilm matrix and may also play a role in biofilm-associated infection.

Striking differences were observed in the MV proteome composition between biofilm and planktonic conditions. Biofilm-derived MVs carry 162 more proteins than MVs from planktonic cultures. 63.9% of proteins identified in biofilm MVs were cytoplasmic, and 29.5% of the proteins were membrane-associated. In contrast, the proteome of MVs purified from MN8 planktonic cultures was comprised of 48.07% cytoplasmic and 45.26% membrane-associated proteins. Consistently, GO analysis of differentially abundant and unique proteins also indicated that cytoplasmic proteins and proteins related to metal ion binding were highly enriched in biofilm-derived MVs, whereas MVs produced from planktonic cultures were enriched in membrane proteins and proteins involved in DNA metabolism, ATP-dependent enzymatic activities, and nucleic acid processing. Bacterial biofilms represent a distinct lifestyle from planktonic cells, as evidenced by their different gene expression profiles (66). The differences in protein expression under the two growth conditions likely contribute to the observed variation in MV protein cargo. Moreover, proteins, such as catalase (67, 68), alkyl hydroperoxide reductase (68), thioredoxin, and flavohaemoglobin (69) contribute to biofilm formation under stress conditions, and these proteins were not detectable in MVs from planktonic cultures. Because MV production by planktonic *S. aureus* is also induced by certain environmental stresses, such as antibiotics, oxidative stress, and nutrient limitation (18), it is tempting to speculate that stress-related proteins associated with MVs play a role in bacterial adaptation.

As a structural component, eDNA interacts with proteins localized within the extracellular matrix and on the bacterial surface via electrostatic interactions (58, 70) to maintain biofilm integrity and structure. Although recent studies have shown that several genes, including *gdpP*, *xdrA, sarA, and apt,* were involved in the release of eDNA within *S. aureus* biofilms (13, 55), the mechanisms by which eDNA is released remain poorly understood. We observed that biofilm-derived MVs carried >10-fold more DNA than MVs from planktonic cultures, and depletion of MVs from biofilm homogenates resulted in a >50% reduction in eDNA levels, indicating that a substantial proportion of eDNA within the biofilm matrix is associated with MVs. We also found that MV-associated eDNA was susceptible to DNase digestion, suggesting that the eDNA is primarily associated with the MV surface rather than encapsulated within the vesicles. These results are consistent with a previous report showing that MVs produced from *S. aureus* planktonic cultures carry DNA that is susceptible to nuclease digestion (26). Similarly, MVs produced from *Pseudomonas aeruginosa* biofilms carry more surface-associated eDNA than those produced under planktonic conditions, and biofilm MV-associated eDNA is susceptible to nuclease treatment (71). Kavanaugh et al. identified >60 eDNA-binding proteins, including cytoplasmic, membrane-associated, and extracellular proteins, in *S. aureus* biofilms and spent medium (15). Many of these proteins, such as Eap, Atl, MntC, PrsA, SaeP, Geh, LtaS, and the PSMβ1 peptide, were also enriched in biofilm-derived MVs. Thus, eDNA may associate with MVs through interaction with these DNA-binding proteins.

Bacterial biofilm formation is a dynamic process, and biofilm dispersal is tightly regulated. Degradation and disruption of the extracellular matrix are essential elements for the dissemination of infection (72). *S. aureus* PSM peptides play a critical role in biofilm remodeling and detachment (56). In addition, enzymes such as proteases and nucleases have been implicated in extracellular matrix degradation and dispersal (70, 73–75). However, the spatial and temporal regulation of these enzymes during biofilm maturation remains poorly understood.

In this study, we purified MVs from drip-flow biofilms cultivated for 48 h, a time point that may precede the dispersal stage. Periasamy et al. used a flow cell system to show that *S. aureus* biofilm dispersal occurred after 72 h (56). Nonetheless, we detected a relatively low abundance of proteases (staphopains A and B), thermonucleases (Nuc1 and Nuc2), and PSMβ1 peptides in biofilm MVs. The expression of these dispersal factors, including proteases, nucleases, and PSMs, is positively regulated by the Agr quorum-sensing system (56), which becomes highly active during late-stage biofilm development. MVs may contribute to biofilm matrix assembly at early stages by delivering key components, including eDNA and proteins. During later stages, matrix-degrading enzymes may become enriched within MVs. Because high concentrations of PSMs can lyse MVs (76), PSMs produced in mature biofilms may disrupt MV structures, thereby facilitating the release of matrix-degrading enzymes and promoting biofilm dispersal. Consistent with this hypothesis, we recovered more MVs from PSM mutant biofilms than from WT biofilms (Fig. S2) using the *S. aureus* MW2 strain, which was previously used to elucidate the role of PSMs in biofilm dispersal (56). Interestingly, in *Candida albicans* biofilms, MVs play multiple roles in biofilm development, such that MV functions are mediated by specific cargo proteins (36). Further studies are needed to elucidate how MV biogenesis and cargo are regulated throughout *S. aureus* biofilm development and how the differentially abundant groups of proteins in MVs influence this process.

## MATERIALS AND METHODS

### Bacterial strains and growth conditions

*S. aureus* strains (listed in S4 Table) were cultivated with aeration in TSB (Difco) or agar plates at 37°C. To grow biofilms, overnight cultures were subcultured into fresh TSB at a 1:1,000 dilution and grown to an OD_650_ of 0.6. Bacterial cells were pelleted by centrifugation at 4,000 × *g* and washed with sterile PBS. The cell pellet was resuspended and further diluted 1:500 in TSB supplemented with 0.2% glucose (TSBG) and inoculated into each chamber of the drip-flow system (BioSurface Technologies Corp), which can hold up to six glass slides (25 mm × 75 mm) with ~125 cm^2^ of surface area. After overnight incubation at 37°C to allow bacterial attachment, the planktonic cells from each chamber were removed from the effluent port, and 10% TSB supplemented with 0.2% glucose was continuously supplied via a peristaltic pump at a flow rate of 0.25 mL/min for 48 h.

### Purification of *S. aureus* MVs from planktonic cultures and drip-flow biofilms

*S. aureus* MN8 was grown in TSB at 37°C to the post-exponential growth phase (OD_650nm_ = 1.2), and MVs from these planktonic cultures were purified as described previously (17, 21, 35). The protein concentrations of purified MVs were determined with Bio-Rad protein dye. MV particles were quantified using a Zetaview Nanoparticle Tracking Analyzer (Particle Metrix) with the following settings: camera sensitivity, 80.0; shutter, 90.0; frame rate, 30 frames per second; temperature, 25°C. The analyses were performed with the built-in ZetaView software 8.02.31.

To purify MVs from *S. aureus* biofilms, the drip-flow biofilms were harvested in sterile PBS at 48 h and disrupted by homogenization. The homogenates were centrifuged at 8,000 × g at 4°C for 30 min to pellet bacterial cells, and the resulting supernatants were filter-sterilized with a 0.45 µm filter. MVs derived from biofilms were pelleted by ultracentrifugation of supernatants at 150,000 × *g* at 4°C for 3 h, followed by density-gradient ultracentrifugation to remove protein aggregates and membrane fragments. Fractions with a similar protein profile on SDS-PAGE were pooled, and the OptiPrep medium was removed by diafiltration with PBS using a Centrifugal Filter Unit (10 kDa, polyether sulfone, Thermo Fisher). Purified biofilm MVs were filter-sterilized, and their protein yield and particle numbers were measured by protein assay and NTA, respectively.

### Proteomic analysis of MVs and biofilm matrix

The supernatants of biofilm homogenates were prepared as described above. After concentration with an Amicon Ultra Centrifugal Filter with 3 kDa MWCO (MilliporeSigma), the protein content was determined. 20 µg of biofilm supernatants, biofilm-derived MVs, or MVs purified from planktonic cultures were loaded onto 10% SDS-PAGE gels and stained with Coomassie blue. Gel sections containing protein bands of each sample were recovered and subjected to a modified in-gel trypsin digestion procedure and analyzed by LC-MS/MS using electrospray ionization and an LTQ Orbitrap Velos Pro ion-trap mass spectrometer (Thermo Fisher Scientific). Peptides were detected, isolated, and fragmented to produce a tandem mass spectrum of specific fragment ions for each peptide. Three biological replicates of each sample were analyzed by LC-MS/MS. Peptide sequences were determined by matching the NCBI Protein database of the *S. aureus* MN8 genome with the acquired fragmentation pattern by the software program SEQUEST (Thermo Fisher Scientific). To remove contaminants and reverse hits, data were filtered to maintain an average 0.8% and 0.1% false discovery rate for protein and peptide identifications, respectively. Protein intensity was quantified as the summation of intensity values (peak height intensity) of individual peptides matched to a given protein. For enhanced confidence in protein identification, individual proteins in each data set were further filtered to maintain a minimum requirement of two total peptides and one unique peptide in at least two of the three biological replicates. Subcellular localization of the proteins was predicted by PSORTb v3.0.3 and UniProt.

Bioinformatic analyses were performed using the R package (v4.1.3). Proteins detected in at least two biological replicates of one experiment group were retained for analysis. Protein intensities were log_2_-transformed to achieve an approximately normal distribution of the data. Due to the high prevalence of group-exclusive protein detections, standard missing-value imputation was not applied. Imputation under these conditions would introduce systematic bias in abundance estimates (77). Therefore, only proteins detected in both conditions were subjected to differential abundance analysis using the limma R package (78), while proteins exhibiting group-exclusive detection patterns were analyzed separately as binary presence/absence calls (79). *P* values were adjusted for multiple comparisons using the Benjamini-Hochberg method to control the FDR, and proteins were considered significantly differentially abundant if they met an FDR < 0.2 (adjusted *P* < 0.2) and an absolute log2 fold change ≥1. Volcano plots were generated by plotting the log2 fold change against the −log10 (adjusted *P* value), and heatmap of differentially abundant proteins were generated to visualize abundance patterns using pheatmap. Missing values were replaced with half the minimum observed intensity per protein for visualization purposes. Correlations in relative protein abundance between biofilm matrix and biofilm-derived MVs were evaluated using the Pearson correlation coefficient (r).

GO over-representation analysis was performed with clusterProfiler using GO annotations retrieved from UniProt. Differentially abundant and unique proteins in MVs from biofilm matrix or planktonic culture were tested separately against the full detected proteome (*n* = 888), which served as the background reference. *P* values were adjusted for multiple testing using the Benjamini-Hochberg (BH) method and GO terms with adjusted *P* < 0.05 were considered significantly over-represented.

### Transmission electron microscopy

Purified *S. aureus* MVs were visualized by TEM as previously described (23). For TEM imaging of samples with fixation, MV samples adsorbed to carbon-coated grids were fixed in 1% (wt/vol) glutaraldehyde in PBS for 5 min before staining with 1% uranyl acetate.

For imaging *S. aureus* biofilm ultrathin sections, the drip-flow biofilm matrix was pelleted by centrifugation at 4000 × g, and the pellet was fixed overnight at 4°C with 2.5% glutaraldehyde/2.5% paraformaldehyde in 0.1 M sodium cacodylate buffer (pH 7.4) and postfixed with 1% osmium tetroxide/1.5% potassium ferrocyanide. After incubation in 1% uranyl acetate, samples were sequentially dehydrated in ethanol (50%, 70%, and 90%) before they were soaked in propylene oxide for 1 h and infiltrated overnight with a 1:1 mixture of propylene oxide and Spurr’s resin. The following day, the samples were embedded in Spurr’s resin and polymerized at 60°C for 48 h. Ultrathin sections were stained with lead citrate and examined in a JEOL 1200EX TEM. Images were recorded with an AMT 2k CCD camera.

### Immunodot blot analysis

*S. aureus* MVs, cell lysates, or serial dilutions of filter-sterilized biofilm homogenates before and after ultracentrifugation were heated at 95°C for 10 min to denature proteins. Samples were applied to nitrocellulose membranes using a 96-well Bio-dot apparatus. The membranes were blocked with 5% skim milk containing rabbit IgG and incubated overnight at 4°C with one of the following primary antibodies: human PNAG monoclonal antibodies (2 μg/mL), CP8 mouse monoclonal antibodies (5A6; 0.5 μg/mL), or LTA mouse monoclonal antibodies (0.5 μg/mL, IBT Bioservices). After washing, membranes were incubated with the appropriate HRP-conjugated secondary antibodies (1:5,000) and developed using a chemiluminescent HRP substrate.

### Confocal microscopy

Biofilms grown in an 8-well chambered cover glass or on glass slides were gently washed 2–3 times with sterile PBS and fixed for 30 min with 4% formaldehyde at room temperature. The biofilms were then rinsed with PBS and stained for 30 min with 5 μM DNA dye SYTO-9 (Thermo Fisher). Imaging was performed with a Nikon NIE upright confocal microscope. Z-stack images were acquired at 1.0 µm intervals through the biofilm depth. The thicknesses (μm) and biovolumes (μm^3^ μm^−2^) of the biofilms were measured using the COMSTAT2 software (www.comstat.dk).

### eDNA measurement

To compare the amount of eDNA associated with MVs produced from drip-flow biofilms and planktonic cultures, DNA was extracted from an equal amount (5 μg) of MVs using a Monarch Spin gDNA Extraction Kit (NEB, MA) according to the manufacturer’s instructions and quantified with a NanoDrop Spectrophotometer (Invitrogen). To assess the proportion of eDNA associated with MVs versus that freely present in the biofilm matrix, equal volumes (30 μL) of filter-sterilized biofilm homogenates collected before (fraction S1) and after MV depletion (fraction S2) by ultracentrifugation were loaded onto a 1% agarose gel and stained with SYBR Safe DNA gel stain. DNA signal intensity was quantified using ImageJ (National Institutes of Health, Bethesda, MD, USA).

In parallel, eDNA levels in serially diluted S1 and S2 fractions were quantified using a HyperLight FluorGreen dsDNA Assay Kit (Fisher) following manufacturer’s instructions. Fluorescence intensity was measured using a plate reader (Infinite 200 Pro, Tecan) with excitation and emission wavelengths of 480 nm and 520 nm, respectively. Background fluorescence from the PBS control was subtracted from each sample reading. eDNA concentration in each sample was calculated from the generated standard curve.

To determine whether free eDNA in the biofilm homogenates could be pelleted during ultracentrifugation, the MV-depleted S2 fractions were further subjected to a second round of ultracentrifugation under the same conditions used for MV isolation. eDNA levels in the resulting supernatants were then quantified using the HyperLight FluorGreen dsDNA Assay Kit (Fisher) according to the manufacturer’s instructions.

## Data Availability

The mass spectrometry proteomics data were deposited in the ProteomeXchange Consortium via the PRIDE partner repository with the data set identifier PXD075156.

## SUPPLEMENTAL MATERIAL

Fig. S1 jb.00210-26-s0001.pdf Biofilm-derived MVs treated with DNase I or proteinase K fail to restore biofilm formation in MN8 static cultures pretreated with the corresponding enzyme.

Fig. S2 jb.00210-26-s0002.pdf MV recovery from drip-flow biofilms formed by S. aureus MW2 and its ∆psma/b/hld mutant strain.

Table S1 jb.00210-26-s0003.xlsx Identified protein list of biofilm-derived MVs, biofilm matrix, and MVs purified from planktonic cultures.

Table S2 jb.00210-26-s0004.xlsx The lipoproteins identified in biofilm matrix and biofilm-derived MVs, respectively.

Table S3 jb.00210-26-s0005.xlsx The shared proteins between biofilm matrix and biofilm-derived MVs.

Table S4 jb.00210-26-s0006.pdf The bacterial strains used in the study.

ASM does not own the copyrights to Supplemental Material that may be linked to, or accessed through, an article. The authors have granted ASM a non-exclusive, world-wide license to publish the Supplemental Material files. Please contact the corresponding author directly for reuse.
