# Supplementary material for: Extracellular membrane vesicles—previously unrecognized components of Staphylococcus aureus biofilms
Source: J Bacteriol. 2026 Aug 7;208(9):e00210-26. doi: 10.1128/jb.00210-26 (PMC13584710; doi:10.1128/jb.00210-26)
Supplement: Supplementary file 1 [file jb.00210-26-s0001.pdf]

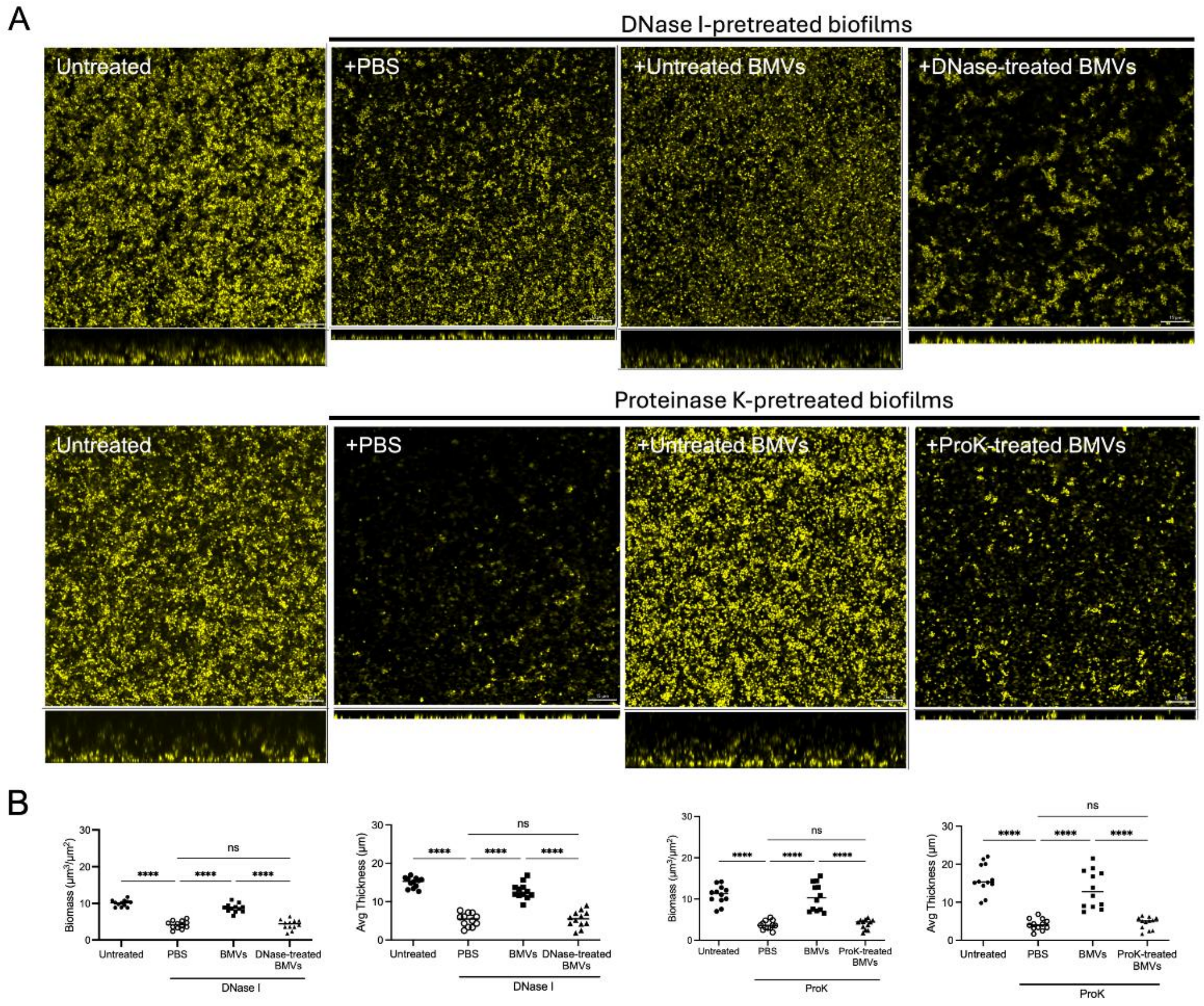

Fig S1. Biofilm-derived MVs treated with DNase I or proteinase K fail to restore biofilm formation in MN8 static cultures pretreated with the corresponding enzyme. **(A–B)** Biofilm-derived MVs (BMVs; 10 µg/ml) were treated with or without DNase I (100 µg/ml) or proteinase K (20 µg/ml) for 1 h and then added to 24-h MN8 biofilms that had been pretreated for 6 h with either proteinase K (20 µg/ml) or DNase I (100 µg/ml), followed by an additional 24 h of incubation. Biofilms from each treatment were imaged by confocal microscopy (A), and biofilm thickness and biomass were quantified from 12 images obtained from three independent biological replicates (B). Data are presented as the mean ± SEM. Statistical significance was determined by one-way ANOVA followed by Tukey's multiple-comparisons test. ****P < 0.0001.
