# Supplementary material for: Extracellular membrane vesicles—previously unrecognized components of Staphylococcus aureus biofilms
Source: J Bacteriol. 2026 Aug 7;208(9):e00210-26. doi: 10.1128/jb.00210-26 (PMC13584710; doi:10.1128/jb.00210-26)
Supplement: Supplementary file 2 [file jb.00210-26-s0002.pdf]

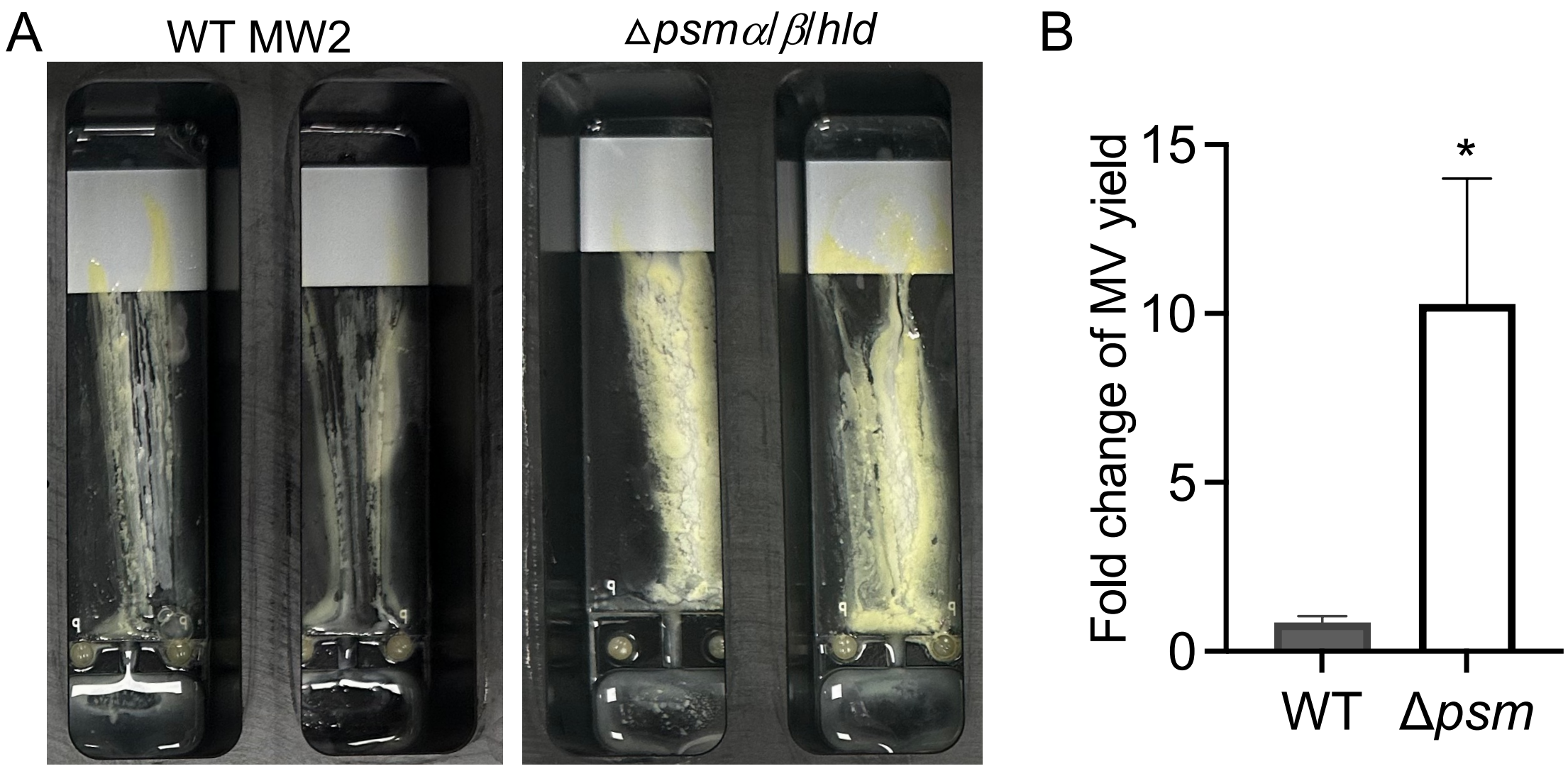

Fig S2. MV recovery from drip-flow biofilms formed by *S. aureus* MW2 and its Δ*psmα/β/hld* mutant strain. **A,** Representative images of drip-flow biofilms formed by WT MW2 and its Δ*psmα/β/hld* mutant after 48 h of growth. **B**, Comparison of MV yield recovered from WT and Δ*psmα/β/hld* mutant biofilms. MV yield was quantified by NTA and normalized to bacterial CFUs for each biofilm. Data are pooled from four independent experiments, presented as mean ± SEM, and analyzed using the Student's *t* test. *, $P < 0.05$.
