# Supplementary material for: Extracellular membrane vesicles—previously unrecognized components of Staphylococcus aureus biofilms
Source: J Bacteriol. 2026 Aug 7;208(9):e00210-26. doi: 10.1128/jb.00210-26 (PMC13584710; doi:10.1128/jb.00210-26)
Supplement: Supplementary file 3 [file jb.00210-26-s0006.pdf]

Table S4. *S. aureus* strains used in this study

Strains	Description[a]	Reference
MN8	MSSA, USA200, ST30, Capsule 8	1
MN8Δ*ica*	MN8 *ica::tet, Tc*[r]	2
MN8Δ*capHIJK*	MN8 *capHIJK*::erm, Em[r]	3
MW2	CA-MRSA, USA400, ST1	4
MW2 Δ*psmα/β/hld*	*psma/psmβ/hld* triple mutant	4
LACΔ*ltaS* suppressor strain	*ltaS::erm; contains additional mutations in gdpp gene;* LTA-deficient strain	5

[a]Tc, tetracycline; Em, erythromycin; MSSA, methicillin sensitive *Staphylococcus aureus*.

References

1. Liu B, Park S, Thompson CD, Li X, Lee JC. 2016. Antibodies to *Staphylococcus aureus* capsular polysaccharides 5 and 8 perform similarly in vitro but are functionally distinct in vivo. *Virulence* **8**, 859–874.

2. Kropec A*, et al.* 2005. Poly-N-acetylglucosamine production in *Staphylococcus aureus* is essential for virulence in murine models of systemic infection. *Infection & Immunity* **73**, 6868–6876.

3. Watts A, Ke D, Wang Q, Pillay A, Nicholson-Weller A, Lee JC. 2005. *Staphylococcus aureus* strains that express serotype 5 or serotype 8 capsular polysaccharides differ in virulence. *Infection and immunity* **73**, 3502–3511.

4. Wang X, Uppu DS, Dickey SW, Burgin DJ, Otto M, Lee JC. 2023. *Staphylococcus aureus* delta toxin modulates both extracellular membrane vesicle biogenesis and amyloid formation. *mBio* **14**, e0174823. PMC10653798

5. Corrigan RM, Abbott JC, Burhenne H, Kaever V, Grundling A. 2011. c-di-AMP is a new second messenger in Staphylococcus aureus with a role in controlling cell size and envelope stress. *PLoS pathogens* **7**, e1002217. PMC3164647
